# Enhancement of aging rat laryngeal muscles with endogenous growth factor treatment

**DOI:** 10.14814/phy2.12798

**Published:** 2016-05-20

**Authors:** Joseph C. Stemple, Richard D. Andreatta, Tanya S. Seward, Vrushali Angadi, Maria Dietrich, Colleen A. McMullen

**Affiliations:** ^1^ Department of Rehabilitation SciencesUniversity of KentuckyLexingtonKentucky; ^2^ Department of PhysiologyUniversity of KentuckyLexingtonKentucky; ^3^ Department of Communication Science and DisordersUniversity of MissouriColumbiaMissouri

**Keywords:** Growth factors, larynx, muscle, neurotrophic, NTF4

## Abstract

Clinical evidence suggests that laryngeal muscle dysfunction is associated with human aging. Studies in animal models have reported morphological changes consistent with denervation in laryngeal muscles with age. Life‐long laryngeal muscle activity relies on cytoskeletal integrity and nerve–muscle communication at the neuromuscular junction (NMJ). It is thought that neurotrophins enhance neuromuscular transmission by increasing neurotransmitter release. We hypothesized that treatment with neurotrophin 4 (NTF4) would modify the morphology and functional innervation of aging rat laryngeal muscles. Fifty‐six Fischer 344xBrown Norway rats (6‐ and 30‐mo age groups) were used to evaluate to determine if NTF4, given systemically (*n* = 32) or directly (*n* = 24), would improve the morphology and functional innervation of aging rat thyroarytenoid muscles. Results demonstrate the ability of rat laryngeal muscles to remodel in response to neurotrophin application. Changes were demonstrated in fiber size, glycolytic capacity, mitochondrial, tyrosine kinase receptors (Trk), NMJ content, and denervation in aging rat thyroarytenoid muscles. This study suggests that growth factors may have therapeutic potential to ameliorate aging‐related laryngeal muscle dysfunction.

## Introduction

Neurotrophins are involved in muscle innervation and differentiation of neuromuscular junctions (NMJs). Clinical studies suggest that age‐related laryngeal motor dysfunction may be of neuronal origin (Baker et al. 1998). In mammals, the neurotrophin family of genes includes: NTF4, neurotrophin 3 (NTF3), neurotrophin 6 (NT6), nerve growth factor (NGF), and brain‐derived neurotrophin factor (BDNF) (Lai and Ip 2003; Huang and Reichardt 2012). BDNF, NTF3, and NTF4 are expressed in skeletal muscle (Gonzalez et al. 1999), and can modulate synaptic efficiency via tyrosine kinase receptors (Trk). TrkB is located at both pre‐ and postsynaptic NMJs (Gonzalez et al. 1999). This localization presents an anatomical medium for the effects of NTF4 on neuromuscular morphology and transmission. NTF4 decreases in rat soleus muscle when neuromuscular transmission is blocked by botulinum toxin (Funakoshi et al. 1995). Inhibition of neuromuscular transmission using botulinum toxin also decreases growth factor expression (Chevrel et al. 2006). NTF4 decreases after sciatic nerve transection (Griesbeck et al. 1995). Increases of neurotrophins have been shown with exercise and contractions induced by sciatic nerve stimulations (Chevrel et al. 2006).

Exogenous NTF4 treatment has been shown to improve neuromuscular transmission in adult rat diaphragm muscle (Mantilla et al. 2004). This improvement is blocked by inhibition of TrkB phosphorylation (Zhan et al. 2003). Neurotrophins may enhance neuromuscular transmission by increasing neurotransmitter release (Park and Poo 2013). NTF4 also increases Nav and voltage‐gated channel conductance (VGCC) leading to an increase in MAPK activation and CREB phosphorylation (Lesser and Lo 1995; Lesser et al. 1997). The long‐term effect of neurotrophins is thought to come from retrograde transport of the Trk complex and their regulation of acetylcholine receptors (AChRs). In sciatic nerve damage, NTF4 treatment reverses the loss of mass in recovering fast muscle fibers (Simon et al. 2004). Other growth factors have successfully been used in humans. Hepatocyte growth factor (HGF), transforming growth factor beta (TGFB), and fibroblast growth factor (FGF) have recently been used in humans for treatment of vocal fold scarring (Hirano et al. 2008, 2012; Branski et al. 2009; Kishimoto et al. 2009). Collectively, these studies point to the promising effects of NTF4 as an injectable therapeutic for treatment of intrinsic laryngeal muscle (ILM) denervation and dysfunction.

Laryngeal muscles contract rapidly and consistently, and are susceptible to the deleterious effects of aging. This activity is thought to contribute to voice problems or dysphagia observed in persons over 65 years of age (Trupe et al. 1984; Ward et al. 1989; Gay et al. 1994; Hagen et al. 1996; Broniatowski et al. 1999; Lundy et al. 1999; Schindler and Kelly 2002). Mechanisms contributing to aging‐related dysfunction include remodeling of the laryngeal mucosa, muscle fiber loss, atrophy, and changes in muscle fiber regeneration (Gambino et al. 1990). ILM atrophy leads to bowing of the vocal folds and inhibiting glottic closure (Sinard 1998; Baker et al. 2001; McMullen and Andrade 2009). Studies in animal models have reported morphological changes consistent with denervation in ILMs, including distal axonal degeneration, smaller endplates, increased variability in endplate architecture, and decreased axon terminals and ACh vesicular clusters (Gambino et al. 1990; Périé et al. 1997; Suzuki et al. 2001; Johnson et al. 2013). We hypothesized that treatment with NTF4 would improve muscle morphology and reduce age‐related denervation in the rat thyroarytenoid muscle.

## Methods

### Animals

Thirty‐two Male Fischer 344‐Brown Norway F1 rats were used for systemic studies. There were eight 6‐mo controls receiving saline osmotic pumps (7‐day or 14‐day) and eight 30‐mo controls used (7‐day or 14‐day). The NTF4 groups consisted of eight NTF4‐treated 6‐mo old (7‐day or 14‐day) and eight 30‐mo old treated rats (7‐day or 14‐day). Twenty‐four rats were used for direct injection. Four rats of each age were used as controls. Eight rats were used in each age group, 6‐ and 30‐mo for direct injections. The age groups selected represent two points in the life span curve of this strain (Turturro et al. 1999). Animals were kept in microisolator cages prior to implantation surgery and given Harlan Teklad food and water ad libitum. Prior to tissue collection, rats were anesthetized with ketamine hydrochloride and xylazine hydrochloride (100 mg/8 mg per kg body weight injected intraperitoneal injection) and killed by exsanguination following a medial thoracotomy. This study was approved by the University of Kentucky Institutional Animal Care and Use Committee (IACUC).

### Osmotic pump implantation

Ophthalmic ointment was applied and the dorsal aspect of the neck was shaved and scrubbed with iodine. Body temperature was maintained with heating pads. Aseptically prepared ALZET^®^ osmotic pumps were filled either with NTF4 or saline, and implanted subcutaneously dorsally on the rat posterior to the scapulae using sterile instruments. First, the pump was inserted into a subcutaneous pocket delivery portal to minimize the interaction between NTF4 and incision healing. A total of 200 ng of NTF4 in 50 ul saline was delivered for either 7‐ or 14 days, based on previously administered dosages for NTF4 and other neurotrophic factors (Simon et al. 2004; Hirano et al. 2008, 2012; Branski et al. 2009; Kishimoto et al. 2009). Meloxicam was administered as a preanesthetic medication and postoperatively for pain relief. Animals were observed in their home cages until they recovered from anesthesia.

### Neurotrophin injection procedure

Direct injections of NTF4 were applied into the rats’ left vocal fold on day one; then, the rats were killed on day seven (Welham et al. 2008). Control rats received a 50 *μ*L direct injection of saline. Rats were sedated with 1–2 mg/kg acepromazine, placed in an induction chamber at 5% isoflurane and 1000 mL/min O_2_ for induction until toe pinch reflex was lost. The rat was reclined in a supine position on a platform, and a specula was used to maintain the oral patency during the procedure. A 50 mm, 30 gauge, 100‐*μ*L syringe (Hamilton) was coupled to a 1.9 mm, 30‐degree endoscope (Storz, Tuttlingen, Germany). This allowed for visualization of the vocal folds and guidance of the syringe. The NTF4 rats received 200 ng in 50 μL saline. This dosage was tolerated with no side effects. The dosage and length of time prior to killing was based on our systemic data and doses determined by other researchers (Simon et al. 2004; Hirano et al. 2008, 2012; Branski et al. 2009; Kishimoto et al. 2009).

### Histology and immunohistochemistry

Larynges were dissected, placed in cryprotectant, embedded in OCT, and frozen in 2‐methylbutane in liquid nitrogen. Cross sections of 10 *μ*m thickness were used to examine the thyroarytenoid muscles. Sections were collected serially; the middle sections on each slide corresponded to the mid‐section of each muscle.

For overall morphology and mitochondrial content, sections were stained with hematoxylin and eosin or with modified Gomori's trichrome (Engel and Cunningham 1963; Sheehan and Hrapchack 1980; McMullen and Andrade 2006). Glycogen content was determined with the Schiff method (Sheehan and Hrapchack 1980). After staining, slides were dehydrated in an ethanol series, cleared with xylene, and mounted in permount. NIH ImageJ software was used to measure the mean fiber area (Rasband 1997‐2012; McMullen and Andrade 2006). Cross‐sectional fiber area was measured at 40× magnification on 3585 fibers. Central nuclei were reported as a ratio of central to total nuclei. Glycogen content was reported as percent of glycogen positive to total fibers from 3936 fibers counted.

To determine denervation, laryngeal sections were fixed with 4% paraformaldehyde, blocked with goat serum, and incubated overnight at 4°C with Nav1.5 (Sigma, St. Louis, MO), followed by an Alexa Fluor conjugated secondary antibody (Invitrogen, Carlsbad, CA). Heart was used as a positive control. Thresholding was based on the labeling intensity of the positive controls. Muscle fibers with >50% labeling were considered denervated (Kulakowski et al. 2011). The ratio of denervated to non‐denervated fibers was calculated from three muscle sections per animal for a total 3630 thyroarytenoid fibers. NMJs were stained with FITC‐labeled *α*‐bungarotoxin (Invitrogen, Carlsbad, CA) and phalloidin, a marker of muscle actin (Invitrogen, Carlsbad, CA) to denote fibers (McMullen and Andrade 2009). We analyzed 59 sections/age from thyroarytenoid muscles.

To determine TrkB intensity and NMJ quantity, sections were prepared as the same for denervation staining, except using antibodies for Trkb (Santa Cruz Biotechnology, Inc, Dallas, TX) and mounted with SlowFade^®^ containing DAPI (Invitrogen, Carlsbad, CA.). Brain tissue was used as a positive control; primary antibody was not added to negative controls. Intensity of TrkB staining was measured with NIH ImageJ software (Rasband 1997‐2012; Collins 2007; Kulakowski et al. 2011). Images were taken at the same light intensity. The background was removed from the images and RGB was measured. Results shown are integrated intensity values for the green color channel (TrkB). We analyzed 58 sections/age.

Sections were imaged with a Nikon E6 microscope equipped with a Digital Sight DS‐U3 camera and Elements software (v 2.0). A total of 20% of histological images were randomly selected and examined by two blinded raters for interrater reliability. Interrater reliability was assessed using the intraclass correlation coefficient (ICC; two‐way, mixed model, single measure). Results demonstrated a high degree of agreement between raters (ICC = 0.934 NMJ counts, 0.943 glycogen counts, 0.913 Nav1.5, 0.965 fiber size, and 0.990 central nuclei counts).

### Data analysis

Results are presented as means and standard error of the mean (SEM). Statistical significance was determined by separate 2 × 2 between‐subjects analysis of variance on the dependent variables of thyroarytenoid fiber size, glycogen content, central nuclei, Nav1.5, TrkB intensity, and NMJ quantity. The independent variables in the systemic group were time and age. The independent variables in the direct injection group were thyroartenoid muscle (left injected side vs. right noninjected side) and age (6‐mo vs. 30‐mo). Significant results were followed up with the Holm‐Sidak Method. Several measures from each animal were averaged for each dependent variable. Animal weight before and after calculations were determined by within analysis of variance. The significance level for rejection of the null hypothesis was set at *P* ≤ 0.05.

## Results

### Fiber size changes with age and treatment

#### Systemic effects

**Figure 1:**
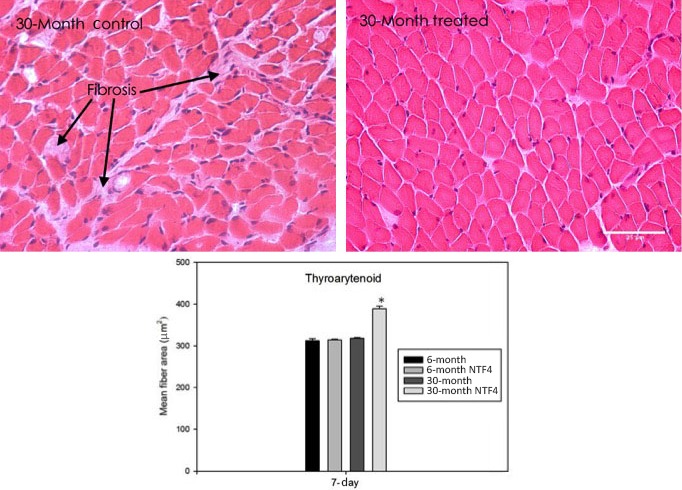
Changes in fiber size and morphology with NTF4 treatment. Representative hematoxylin stained sections of NTF4 7‐day treated (right) and untreated (left) thyroarytenoid muscles at 30‐mo of age. There is an increase in fiber size from control to treated. Treatment with NTF4 changes the 30‐mo fiber size to that of a younger 6‐mo old animal. *P < 0.05 versus 30‐mo. (scale bar = 25 *μ*m), *P *<* *.001). Arrows in the top picture point to an area of fibrosis. There is also a qualitative decrease of fibrosis in the treated animals.

Systemic NTF4 application produced significant differences in muscle fiber area (Fig. 1). (See Table 1 for systemic statistics). In the 7‐day systemic NTF4 group, mean muscle fiber area significantly increased at 30‐mo compared to controls. In the 14‐day systemic NTF4 group, thyroarytenoid muscle fiber area showed no change at 30‐mo compared to controls [*F*(7, 28) = 4.480, *P *<* *0.001]. There was no effect of treatment on body weight (*P* < 0.05).

**Table 1:** Systemic NTF4 statistics

	6‐mo control	6‐mo NTF4 treated	30‐mo control	30‐mo NTF4 treated
7‐Day Treatment Group				
*n* = 4 per group				
Fiber size (*μ*m^2^)	312.51 ± 5.43	314.46 ± 1.89	318.66 ± 2.17	389.03 ± 6.12^a^
Central nuclei (% central to total nuclei)	2.86 ± 0.21	3.37 ± 0.19	1.37 ± 0.15	4.16 ± 0.15
Nav1.5 (% as total to positive fibers)	28.74 ± 1.19	10.74 ± 1.9^a^	35.94 ± 1.42	8.42 ± 1.35^a^
NMJ quantity	42.33 ± 2.11	47.67 ± 5.30	29.5 ± 2.65	27.67 ± 0.62
PASH counts (Glycogen content)	16.83 ± 0.53	0.01 ± 0.003^a^	6.60 ± 0.17	6.38 ± 0.50^a^
TrkB intensity	6.45 ± 2.93	10.92 ± 2.05^a^	25.71 ± 3.66	13.09 ± 2.32^a^
Average animal weight before (grams)	397.75 ± 29.83	380.75 ± 21.78	582.00 ± 60.60	571.50 ± 67.42
Average animal weight after (grams)	387.00 ± 29.71	385.25 ± 50.76	577.50 ± 50.73	564.00 ± 65.82
14‐Day Treatment Group				
*n* = 4 per group				
Fiber size (*μ*m)	434.79 ± 9.51	415.25 ± 8.33	425.45 ± 5.07	423.84 ± 4.61
Central nuclei (% central to total nuclei)	5.57 ± 0.71	4.80 ± 1.02	5.29 ± 0.35	4.90 ± 0.28
Nav1.5 (% as total to positive fibers)	21.22 ± 7.53	15.35 ± 1.51^a^	21.55 ± 0.85	13.73 ± 1.49^a^
NMJ quantity	28 ± 1.16	45.25 ± 2.14^a^	30.25 ± 5.45	33.67 ± 3.27
PASH counts (Glycogen content)	0 ± 0	10.16 ± 0.38^a^	4.35 ± 0.17	9.09 ± 0.11^a^
TrkB intensity	4.43 ± 1.89	30.53 ± 0.01^a^	34.54 ± 0.01	43.33 ± 3.93^a^
Average animal weight before (grams)	329.75 ± 21.23	371.25 ± 15.11	616.25 ± 65.66	585.00 ± 18.85
Average animal weight after (grams)	400.75 ± 22.17	375.25 ± 17.31	580.50 ± 81.28	579.25 ± 19.36

a Significantly different from control.

#### Direct injection effects

(See Table 2 for direct injection statistics): The effects of direct injection of NTF4 into the thyroarytenoid muscles were determined 7‐days post treatment. With direct NTF4 injection, muscle fiber area at 30‐mo was significantly decreased compared to controls [*F*(7, 24) = 5.672, *P *<* *0.001].

**Table 2:** Direct injection statistics

	6‐mo control *n* = 4	6‐mo NTF4 injected *n* = 8	30‐mo control *n* = 4	30‐mo NTF4 injected *n* = 8
Fiber size (*μ*m)	328.95 ± 18.55	331.95 ± 12.22	350.64 ± 22.54	322.61 ± 8.51^a^
Central nuclei (% central to total nuclei)	7.34 ± 0.49	3.87 ± 0.32^a^	5.39 ± 0.38	7.95 ± 2.42
Nav1.5 (% as total to positive fibers)	3.75 ± 2.35	6.08 ± 0.10	22.65 ± 0.47	9.65 ± 0.25^a^
NMJ quantity	9.00 ± 3.0	35.67 ± 10.71^a^	8.13 ± 1.93	11.5 ± 0.87^a^
PASH counts (Glycogen content)	2.96 ± 1.66	7.38 ± 2.47	5.93 ± 1.21	5.25 ± 0.41
Average animal weight before (grams)	370.65 ± 5.85	360.95 ± 5.85	554.60 ± 1.40	599.40 ± 95.70
Average animal weight after (grams)	373.05 ± 5.05	366.7 ± 7.20	565.45 ± 10.05	602.50 ± 92.9

a Significantly different from control.

### Muscle regeneration

#### Systemic effects

In the 7‐day systemic NTF4 group, thyroarytenoid mean percent of central nuclei increased for the 30‐mo group compared to aged controls, although the effects did not reach significance. In the 14‐day systemic NTF4 group, regeneration effects were not observed in the 30‐mo animals compared to controls. There was a significant main effect of treatment for groups between the 7‐ and 14‐day treatment groups as a whole [*F*(7, 24) = 11.22, *P *<* *0.001].

#### Direct injection effects

With direct NTF4 injection, mean percent of central nuclei decreased significantly in the 6‐mo injected group compared to controls. Central nuclei displayed an increasing trend for the 30‐mo group compared to controls, although this effect did not reach significance. There was a main effect of treatment [*F*(7,15) = 1.76, *P *<* *0.001)].

### Glycogen content

#### Systemic effects

**Figure 2:**
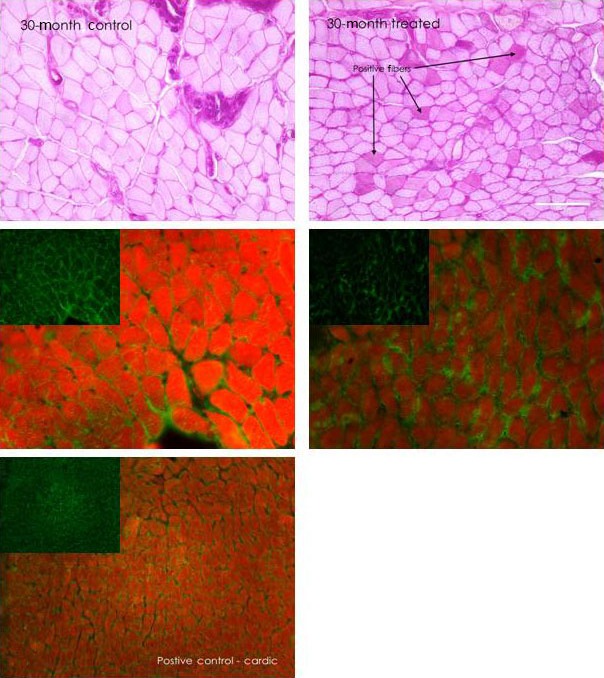
Changes in aerobic capacity and innervations with NTF4 treatment. Representative glycogen (top) and Nav1.5 (bottom) stained sections from NTF4 14‐day treated (right) and untreated (left) thyroarytenoid muscles at 30‐mo of age. Increase of glycogen‐positive muscle fibers indicates a change in respiratory capacity; darker pink fibers are considered glycogen positive. Reduction of denervation with age as measured by Nav1.5 labeling (green) and phalloidin to denote fibers (red). Left middle panels are representative Nav1.5 (green) and phalloidin (red) stained sections from untreated thyroarytenoid muscles. The right middle panels are treated muscles. Green insert is Nav1.5 staining alone. Notice decrease of fibers stained for Nav1.5 after treatment with NTF4. Bottom left micrograph is a positive control consisting of heart muscle from a 6‐month control animal (scale bar = 25 *μ*m), (*P *<* *.001).

In the 7‐day systemic NTF4 group, percent thyroarytenoid glycogen‐positive fibers significantly decreased for the 6‐ and 30‐mo groups compared to aged controls. In the 14‐day systemic NTF4 group, percent thyroarytenoid glycogen‐positive fibers significantly increased for the 6‐ and 30‐mo groups compared to aged controls [*F*(7, 28) = 6.209, *p *<* *0.001] (Fig. 2).

#### Direct injection effects

There was a trend in both treated groups of an increase of glycogen‐positive fibers although the effects did not reach significance [*F*(7, 25) = 0.789, *P *<* *0.603].

### Denervation and neuromuscular changes

#### Systemic effects

**Figure 3:**
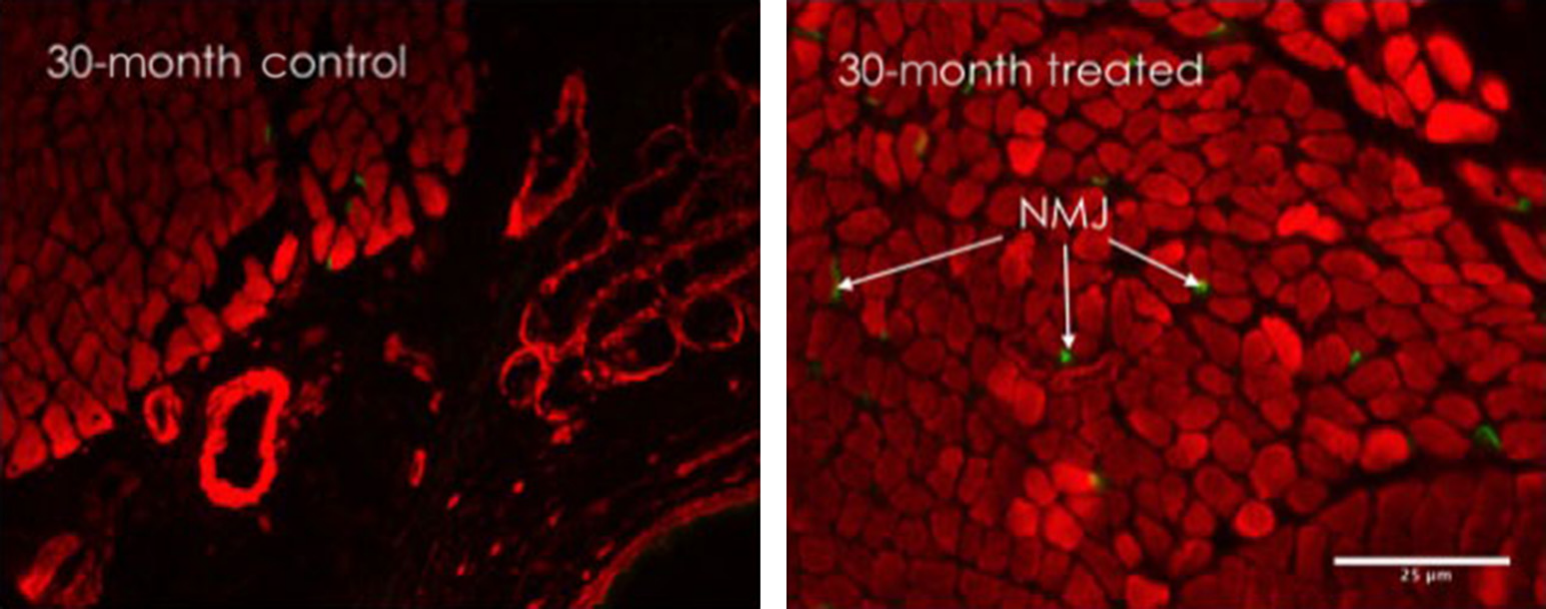
Changes in NMJs with NTF4 treatment: Representative fluorescence microscopy images of NMJs from NTF4 14‐day treated thyroarytenoid muscle sections from different animals labeled with *α*‐bungarotoxin (green) and phalloidin (red) showing that NMJ number increases with NTF4 treatment at 30‐mo (scale bar = 25 *μ*m), (*P *<* *.001).

In the 7‐and 14‐ day systemic NTF4 groups, the percent of thyroarytenoid‐denervated fibers as measured by Nav1.5, significantly decreased for the 30‐mo group compared to aged controls [*F*(7, 17) = 25.08, *P *<* *0.001]. At 6‐mo, there was a significant increase in NMJ quantity between the control and treated at 14‐days. In the 7‐ and 14‐ day systemic NTF4 groups, thyroarytenoid mean NMJ quantity did not change significantly at 30‐mo, but there was a main effect of treatment [*F*(7, 28) = 7.33, *P *<* *0.001]. This may be due to a change in size of the muscle fiber area (Fig. 3).

#### Direct injection effects

With direct NTF4 injection, the percent of thyroarytenoid denervated fibers significantly decreased for the 30‐mo group compared to aged controls [*F*(7,15) = 14.71, *P *<* *0.001]. There was a significant increase in NMJ quantity in the 30‐mo injected animals. There was also a main effect of treatment [*F*(7, 28) = 6.79, *P *<* *0.001].

### Mitochondria content

**Figure 4:**
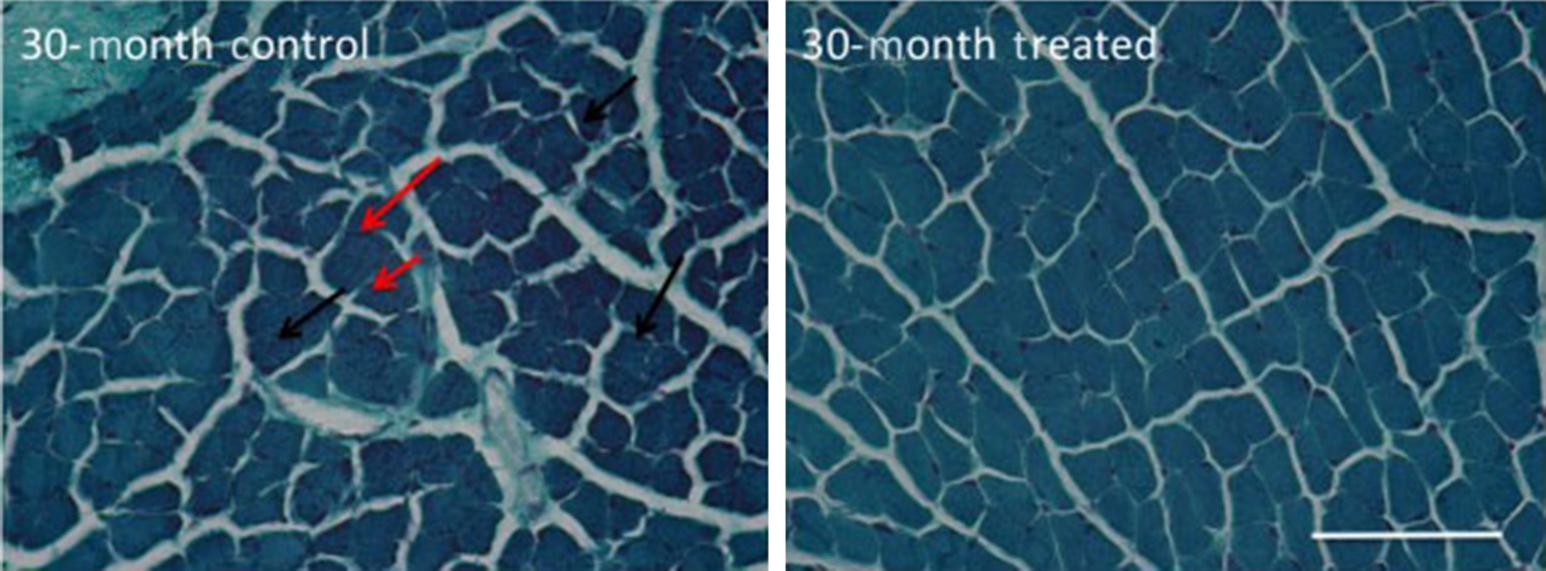
Evidence of red ragged fibers in aging thyroarytenoid muscle and changes with NTF4 treatment. Representative Gomorori's trichrome images for overall mitochondria content from control 30‐mo (left) and 14‐day NTF4‐treated muscles (right). There appear to be more mitochondria clusters as (denoted by black arrows) and red ragged fibers (denoted by red arrows) in untreated aging muscle (scale bar = 25 *μ*m).

Ragged red fibers were not found in 6‐mo controls or in any NTF4‐treated thyroarytenoid muscles, suggesting stable aerobic capacity with systemic and direct treatment (Fig. 4).

### TrkB intensity

**Figure 5:**
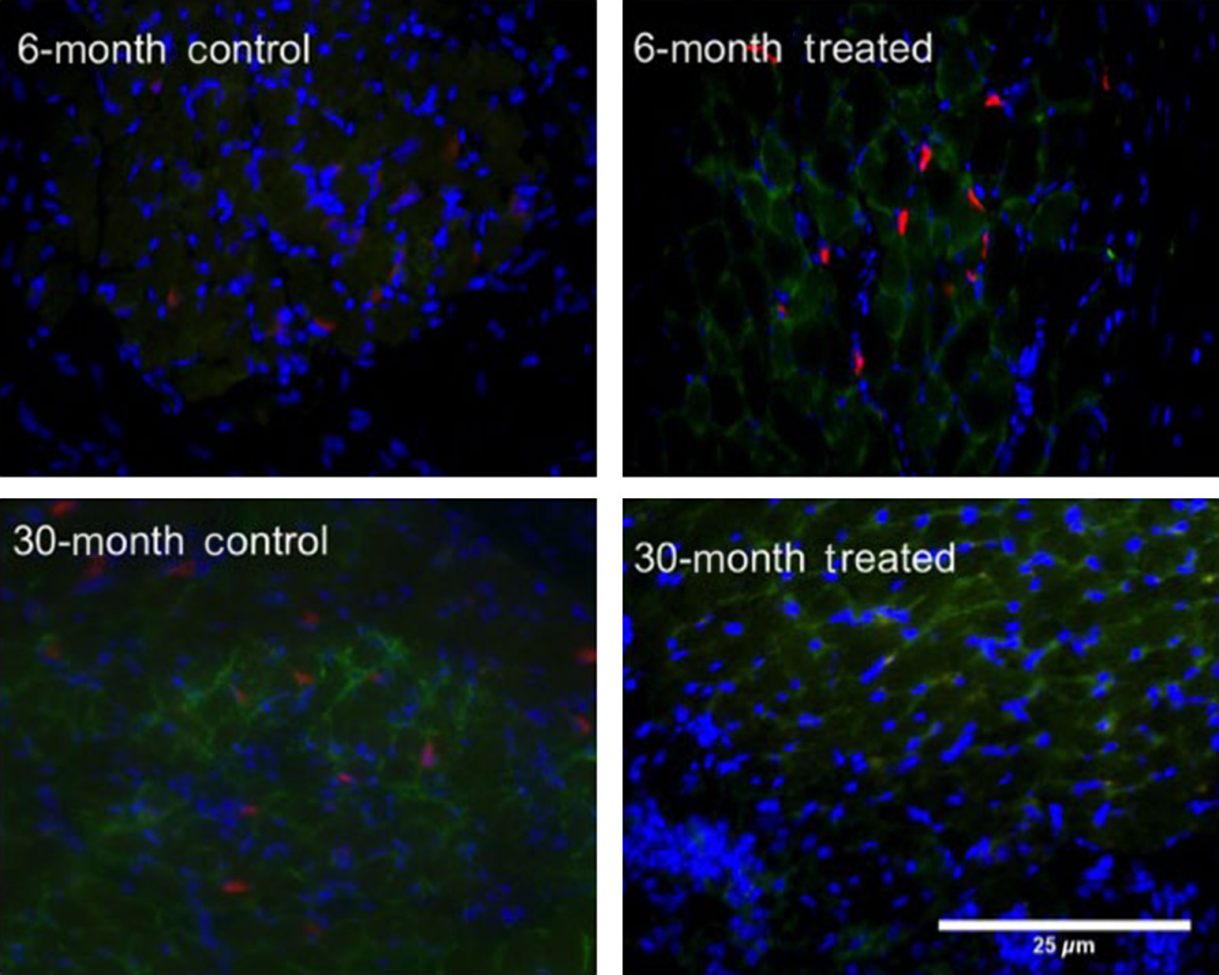
TrkB intensity changes with NTF4 treatment. Representative fluorescence microscopy images of TrkB (Green), DAPI (Blue), and *α*‐bungarotoxin (Red) from NTF4 14‐day control (Left panel) and 14‐day treated thyroarytenoid muscle sections (Right panel) showing that TrkB intensity number increases with NTF4 treatment at 6‐ and 30‐mo (scale bar = 25 *μ*m), (*P *<* *.001).

In the systemic group, there was a decrease in TrkB intensity with treatment in the NTF4 7‐day 30‐month group. Conversely, in the 14‐day group, there was an increase in TrkB intensity with treatment at 30‐mo compared to control [*F*(7, 28) = 31.26, *P *=< 0.001)] (Fig. 5).

## Discussion

These data provide the first evidence for the effectiveness of neurotrophins to induce ILM remodeling responses. We hypothesized that aging ILMs would change following treatment with NTF4. Changes found in oxidative, metabolic, and glycolytic capacity are consistent with fast‐contracting and fatigue‐resistant fiber types. A decrease in mean fiber area in the muscle after direct neurotrophin treatment is similar to changes in other skeletal muscles after endurance training.

The study of ILMs is important to develop effective interventions or preventative measures in the aging human. Previous studies have documented alterations in aging ILMs, including deceases in fiber size, mass, total number of fibers present, regenerative capacity, NMJ size/quantity, changes in myosin heavy chain isoforms, and contractile function (Hagen et al. 1996; McMullen and Andrade 2006, 2009; Kulakowski et al. 2011; Nishida et al. 2013). The purpose of this study was to determine if application of neurotrophins could ameliorate muscle deterioration associated with age in the ILM.

Our results demonstrated an age‐associated increase in fiber size in the thyroarytenoid muscle after 7 days of systemic NTF4 administration. Previous data show that aging rat muscle fibers change in the opposite direction compared to the muscles that were treated systemically with NTF4 (McMullen and Andrade 2006). With direct injection of NTF4, fiber size decreased at 30‐mo of age, similar to what we have previously observed after laryngeal nerve stimulation (McMullen et al. 2011). The time and dosage administration results suggest the need for further investigation to help explain fiber size change differences observed between systemic and direct application of NTF4.

With application of neurotrophins in our study, there is a qualitative decrease in the appearance of fibrosis (Fig. 1). Fibrosis is a diminution of muscle quality due to an increase of fat and other noncontractile materials (Serrano et al. 2011). The aging process intensifies the fibrotic phenotype. In normal fibers, the nuclei are located in the periphery. A central nucleus within a fiber represents a nonspecific marker of muscle damage, such as fibrosis, and/or regeneration. Central nuclei are frequently seen in dystrophic muscle and during development (Banker and Engel 2004). With direct NTF4 treatment, the decrease or lack of change of central nuclei suggests a decrease in regenerative capacity. However, the nonsignificant effect for central nucleation in the systemic NTF4 across age groups in thyroarytenoid may indicate different regeneration capacities with treatment. The diminished regenerative capacity of the thyroarytenoid could be related to a lack of autophagy or impaired satellite cell function (McLoon and Wirtschafter 2003; McMullen et al. 2009). Alternatively, other researchers have shown that the thyroarytenoid regenerates consistently throughout the lifespan to compensate for fiber loss related to disease or injury, although this capacity appears to decrease with advancing age (Lee et al. 2012). The static regenerative capacity in the thyroarytenoid muscle may be detrimental to key functions of the larynx including respiration, swallowing, and voice and airway protection in the elderly. Another possibility is that these aging muscles do not need to regenerate based on the physiological differences between the muscles (McLoon and Wirtschafter 2003; McMullen et al. 2009).

Others and we have demonstrated that aging ILMs display functional evidence of denervation (Périé et al. 1997; Johnson et al. 2013). A decrease in NMJ abundance would demonstrate a loss of morphologically defined innervation points in these muscles. We have previously demonstrated that NMJs become smaller and less abundant in aging ILMs (Périé et al. 1997). In systemically NTF4‐treated thyroarytenoid muscle, NMJ quantity increased in the 6‐mo 14‐day treated group compared to control. Our current data also show that NTF4 systemic treatment significantly reduced denervation for the 30‐month animals as measured by Nav1.5 labeling in both systemic and direct injection groups. NTF4 may thus be effective for the reinnervation of aging ILMs due to enhanced muscle morphology. Changes in NMJs and innervation can also be due to a loss of fast motor units that can occur with normal aging (Jang and van Remmen 2011). It will be beneficial to determine if contractile function in the ILMs improves and motor unit number increases with NTF4 treatment.

Intracellular glycogen content at 6‐mo accounts for ~7% of muscle fibers, with the proportion of glycogen‐positive muscle fibers significantly increasing at 30‐mo to 27% (McMullen and Andrade 2006), thus indicating a shift in the muscle cell respiratory physiology with age (Fig. 2). With NTF4 treatment, glycogen‐positive thyroarytenoid muscle fibers decreased at 6‐ and 30‐mo for the 7‐day treatment groups. Alternatively, in the 14‐day NTF4 treatment groups, 6‐mo and 30‐mo animals showed an increase of glycogen‐positive muscle fibers indicating an overall shift in respiratory capacity. This implies a differential effect as a factor of treatment time. Both ages of the direct injection groups also showed a trend in increasing glycogen content. These data suggest an interaction effect between changes in muscle aerobic capacity and treatment duration. Muscles require different isoenzymes at different ages. Similar results to the 14‐day group finding have been shown in a previous publication where we electrically stimulated the recurrent laryngeal nerve (McMullen et al. 2011). If there is a reduction in glycogen, then there will be an impairment of glycogen breakdown needed for muscle metabolism. Depletion of glycogen can impair metabolism and have a negative effect on performance. The 14‐day systemic group may have a better response in terms muscle aerobic capacity. It is possible that NTF4 may have augmented glycogen loading (Garvey et al. 2015). We did not examine the content of glycogen intermediates such as maltopentaose or maltotriose. In future experiments, it would be interesting to test the effects of NTF4 application in rats with demonstrated alterations in glycogen transport or metabolism.

An additional qualitative observation demonstrated changes in mitochondrial content (Fig. 4). These changes have been associated with alterations in muscle fiber size due to fiber atrophy and fiber loss (Hepple et al. 2004; Short et al. 2005). In aging ILMs, mitochondrial dysfunction may also be an important age‐related event. Aging thyroarytenoid muscles from 30‐mo old rats contain fibers with abnormally large mitochondrial accumulations (ragged red fibers), representing 10.4% (±4.5) of counted muscle fibers (McMullen and Andrade 2006). These qualitative findings are suggestive of diminished aerobic capacity. Ragged red fibers were not found in 6‐mo controls or in any NTF4‐treated thyroarytenoid muscles, suggesting stable aerobic capacity for these muscles with treatment.

Finally, we examined the expression of Trkb (Fig. 5) and found differential effects in TrkB with systemic NTF4 treatment in both the 7‐and 14‐day groups. It has been suggested that TrkB expression is reduced at the NMJ with age (Personius and Parker 2013). These experiments were conducted in soleus muscle, which is composed of mostly slow muscle fibers, whereas the ILMs are mostly fast muscle fibers (Conner et al. 2002). It has been proposed that TrkB may take part in the maintenance and protection of sensory structures in the laryngeal mucosa (Yamamoto et al. 2011). The appearance of higher levels of endogenous TrkB and its increase with treatment suggests the possible enhancement of NMJ transmission with systemic treatment.

## Conclusion

This study examined the effects of neurotrophin application in aging rat ILMs. These data display the effect of neurotrophin use to induce laryngeal remodeling responses in an animal model. Changes in oxidative, metabolic, and glycolytic capacity are consistent with fast‐contracting and fatigue‐resistant fiber type. Future study is needed to examine the functional effects of these neurotrophins on the aging laryngeal muscle. This study demonstrates that neurotrophins may have therapeutic potential on aging‐related laryngeal muscle dysfunction.

## Conflict of Interest

None declared.
